# MALT Lymphoma of the Tongue in a Patient with Sjögren’s Syndrome: A Case Report and Literature Review

**DOI:** 10.3390/diagnostics11091715

**Published:** 2021-09-19

**Authors:** Jiyeon Baik, Hye-Jin Baek, Kyeong-Hwa Ryu, Hyo-Jung An, Seulki Song, Ho-Joon Lee, Yeonah Kang

**Affiliations:** 1 Department of Radiology, Haeundae Paik Hospital, Inje University College of Medicine, 875 Haeun-Daero, Haeundae-Gu, Busan 48108, Korea; jbaik6@gmail.com (J.B.); hojoon.lee@paik.ac.kr (H.-J.L.); bsb2312@gmail.com (Y.K.); 2 Department of Radiology, Gyeongsang National University Changwon Hospital, Gyeongsang National University School of Medicine, 11 Samjeongja-Ro, Seongsan-Gu, Changwon 51472, Korea; ryukh0329@gmail.com; 3 Institute of Health Sciences, Gyeongsang National University School of Medicine, 816-15 Jinju-Daero, Jinju 52727, Korea; ariel2020@naver.com; 4 Department of Pathology, Gyeongsang National University Changwon Hospital, Gyeongsang National University School of Medicine, 11 Samjeongja-Ro, Seongsan-Gu, Changwon 51472, Korea; 5 Department of Otorhinolaryngology, Gyeongsang National University Changwon Hospital, Gyeongsang National University School of Medicine, 11 Samjeongja-Ro, Seongsan-Gu, Changwon 51472, Korea; ssgi87@naver.com

**Keywords:** Sjögren’s syndrome, MALT, lymphoma, tongue, minor salivary gland, CT, MRI

## Abstract

Sjögren’s syndrome (SS) is a systemic chronic autoimmune disorder characterized by lymphocytic infiltration of the exocrine glands, as well as oral and ocular dryness. Among the late complications, malignant lymphoma is the most serious complication of SS. The risk of lymphoma in patients with SS has been estimated to be approximately 7–19 times higher than that in a generally healthy population. Although various histologic subtypes of lymphoma can occur in patients with SS, mucosa-associated lymphoid tissue (MALT) lymphoma accounts for 48–75% of malignant lymphomas that are frequently located in the parotid gland. However, MALT lymphoma affecting the tongue in patients with SS is extremely rare. Here, we share our experience with a unique case of MALT lymphoma of the tongue, originating from the minor salivary gland tissue in a patient with SS. Through this case report, we emphasize that MALT lymphoma should be considered in the differential diagnosis of a tongue mass in patients with SS.

## 1. Introduction

Sjögren’s syndrome (SS) is an autoimmune disease characterized by lymphocytic infiltration of the exocrine glands, such as the salivary and lacrimal glands. In addition, immune abnormalities, such as the production of various autoantibodies, including anti-SS-A and SS-B antibodies, and increased levels of polyclonal immunoglobulin are also observed. As the exocrine glands are destroyed, the production of saliva and lacrimal fluid is reduced, causing dry eyes and mouth. This condition is clinically diagnosed when specific serum autoantibodies are detected. Patient prognosis is affected by complications, and malignant lymphoma is one of the most severe complications [1]. The risk of lymphoma in patients with SS has been estimated to be approximately 7–19 times higher than the expected incidence in a generally healthy population [2]. Approximately 5% of patients with SS develop B-cell non-Hodgkin lymphoma (NHL), and the majority are mucosa-associated lymphoid tissue (MALT) lymphomas, which are commonly encountered in the parotid gland. However, MALT lymphomas are rarely observed in the minor salivary glands [3,4,5,6]. To the best of our knowledge, this is the first report of a MALT lymphoma occurring in the minor salivary gland of the tongue in a patient with SS. Herein, we present a case of lingual MALT lymphoma in a patient with SS, along with the radiological and histopathological findings. We also performed a review of the relevant literature regarding this disease for the accurate diagnosis and appropriate treatment of patients with SS with a tongue mass.

## 2. Case Report

This was an observational case study that did not alter the patient’s management and clinical outcomes. Thus, ethical approval was not required for this case report, and patient consent was waived due to the retrospective nature of the study.

**Figure 1:**
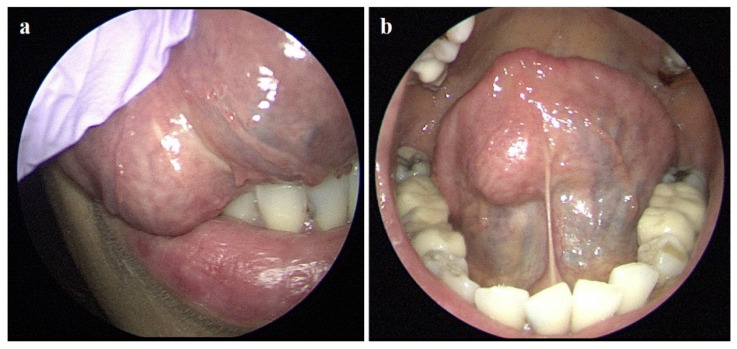
Laryngoscopy (**a**,**b**) shows a firm and fixed nodule in the right oral tongue (3 cm in maximum diameter). The overlying mucosa is intact; therefore, the nodule is considered as a submucosal lesion.

**Figure 2:**
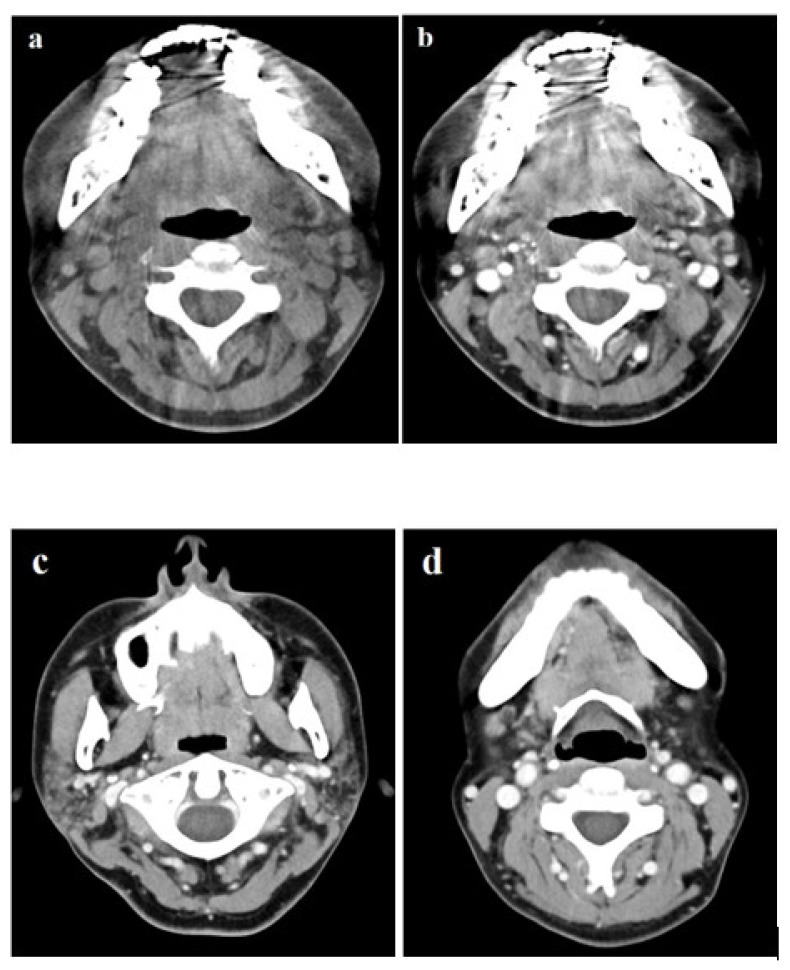
The lesions are not visible on non-enhanced axial (**a**) and enhanced axial CT (**b**) images owing to the presence of severe beam hardening artifacts by dental prosthesis. The contrast-enhanced CT (**c**) image shows multiple granulonodular opacities with partially fatty and mildly atrophic changes in both parotid glands. The contrast-enhanced CT (**d**) shows total fatty degeneration of both submandibular glands, however, glandular volume is conserved.

**Figure 3:**
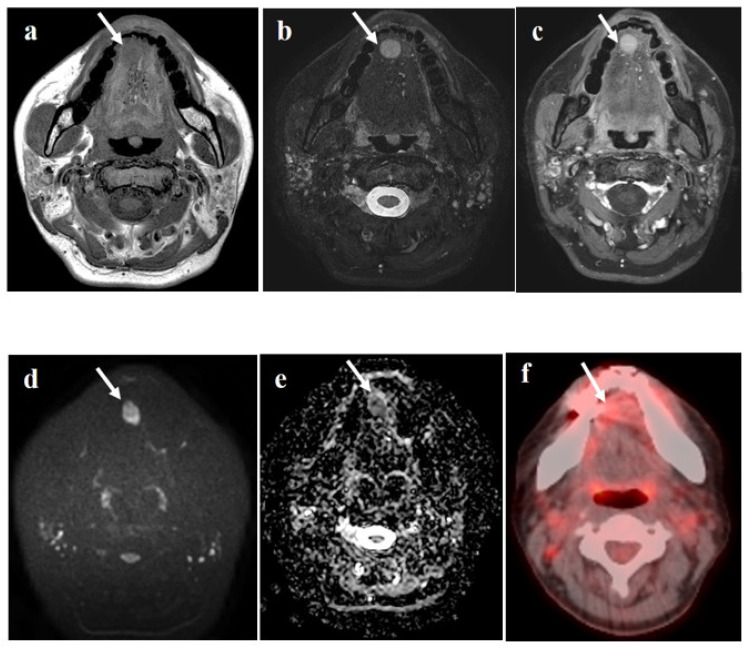
On T1-weighted image (**a**), the nodule is isointense with partially defined. On T2-weighted image (**b**), the nodule reveals omogeneously high signal intensity. The lesion shows a homogeneous enhancement on the contrast-enhanced T1-weighted image (**c**). The nodule showed diffusion restriction on diffusion-weighted image and ADC map (**d,e**). On 18-F FDG PET/CT image (**f**), the nodule shows mild FDG uptake with SUVmax of 4.8 g/mL.

A 49-year-old woman visited the ENT clinic for a painless mass with a foreign body sensation in the tongue for 2 weeks. Physical and laryngoscopic examinations revealed a well-defined, hard, fixed mass, approximately 3 cm in size, with intact overlying mucosa on the inferior surface of the right side of the tongue (Figure 1a,b). The patient had no other underlying diseases diagnosed to date, and the initial laboratory results showed a white blood cell count slightly decreased from the normal levels (2.4 × 10^9^/L). Computed tomography (CT) was performed using a dual-layer detector CT unit (Iqon Spectral CT, Philips Healthcare, Best, the Netherlands) to evaluate the tongue mass, but it was difficult to identify the lesion because of metallic dental artifacts (Figure 2a,b). However, we observed chronic sialadenitis with mild atrophy of the bilateral parotid glands, showing inhomogeneous glandular attenuation and heterogeneous enhancement with multiple granulonodular opacities, indicating a high probability of SS (Figure 2c,d). There was no cervical lymphadenopathy or other remarkable findings in the neck. To delineate and characterize the tongue mass, neck magnetic resonance imaging (MRI) was performed one week later using a 3T system with a 20-channel head and neck coil (Ingenia 3.0 CX; Philips Healthcare, Best, the Netherlands). On MRI, the lesion appeared as a well-defined, homogeneously enhancing nodule with homogeneous T1 hypointensity and T2 hyperintensity (Figure 3a–c). The lesion also showed diffusion restriction without intralesional hemorrhage (Figure 3d,e), and there was no evidence of invasion into the surrounding tissue plane. Additional 18F-fluorodeoxyglucose positron emission tomography/computed tomography (18F-FDG PET/CT) was performed for staging because of the possibility of malignancy. The lesion showed mild FDG uptake with a maximum standardized uptake value of 4.8 g/mL (Figure 3f), and there was no other FDG uptake within scan range on 18F-FDG PET/CT. During the preoperative diagnostic work-up, serologic tests to evaluate Sjögren’s syndrome were also performed as follows: the anti-nuclear antibody (ANA) and anti-SS-A (Ro) antibody test results were positive with a titer of 1:1280 (normal range: <1:80) for ANA antibody and with a level of >200 U/mL (normal range: <15 U/mL) for anti-SS-A (Ro) antibody, whereas the anti-SS-B (La) antibody and rheumatoid factor (RF) test results were negative.

**Figure 4:**
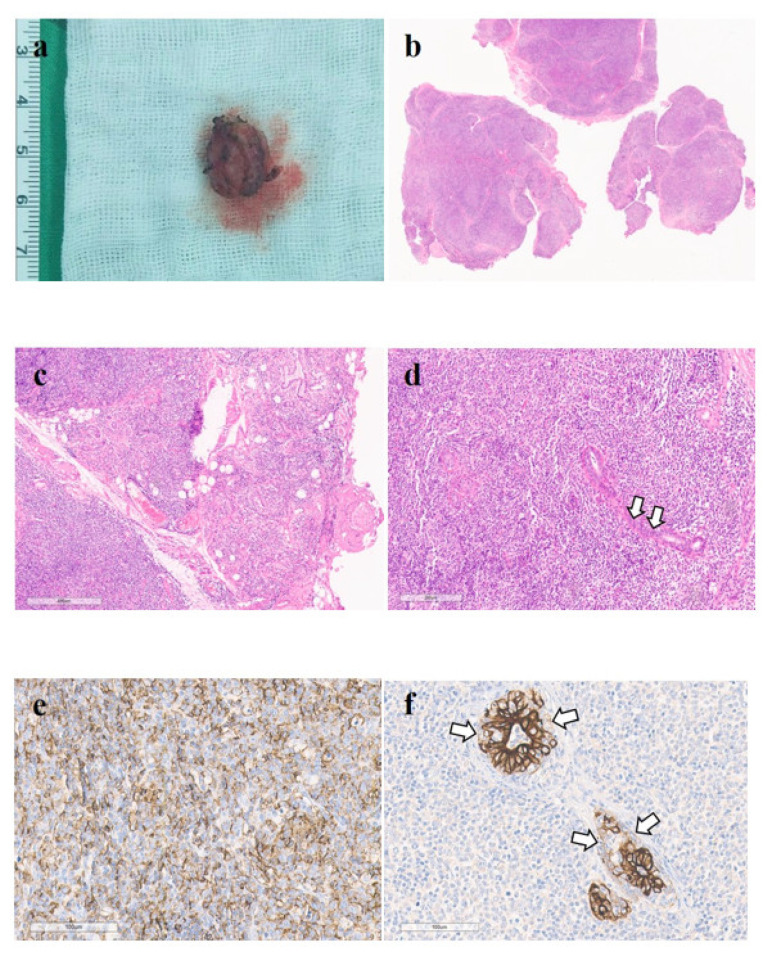
(**a**) A photograph of the specimen shows the excised nodule (2.5 cm in size). (**b**) Extensive lymphocytic infiltration of the minor salivary gland is noted. There are multiple lymphoid follicles with germinal centers (hematoxylin and eosin, ×10). (**c**) In the periphery, minor salivary glandular tissue with residual intercalated ducts appears fibrotic and infiltrated by numerous lymphoid cells. Seromucinous units or mucous acini of the minor salivary glands are hardly seen (×80 hematoxylin and eosin). (**d**) On higher magnification, the lymphoid infiltration appears to be intense, and monotonous atypical lymphoid cells are visible as well. Lymphoepithelial lesions (arrows) were visible, and mucosa-associated lymphoid tissue (MALT) lymphoma was suspected (×100, hematoxylin and eosin). (**e**) These monotonous atypical-looking lymphoid cells were diffusely positive for CD20. B-cell phenotype lymphoma was suspected (CD20, ×200). (**f**) Salivary gland ductal cell infiltration by cytokeratin-negative atypical-looking lymphoid cells indicates the presence of a lymphoepithelial lesion. Extranodal marginal zone B-cell lymphoma of MALT is highly suspected (cytokeratin, ×200).

The patient underwent an excisional biopsy of the mass. The frozen section of the mass showed a soft tissue nodule 1.2 × 0.7 × 0.5 cm in size. The histopathological findings demonstrated the nodular proliferation of atypical lymphoid cells containing lymphoepithelial lesions and destruction of the atypical glandular architecture around the lesion (Figure 4a–d). The immunohistochemical staining results showed atypical lymphoid cells expressing BCL-2, BCL-6, Pan T (CD3), CD4, CD8, Pan B (CD20), and CD79a, and cytokeratin was also observed in the lymphoepithelial lesions (Figure 4e,f). However, negative results were observed for CD10, CD56, and cyclin D1 immunohistochemical staining. In addition, immunoglobulin heavy chain gene rearrangement was also detected. Based on these results, the mass was confirmed to be SS-associated MALT lymphoma of the minor salivary gland of the tongue. The final stage was diagnosed as Ann Arbor stage IE.

After the histopathological diagnosis of the tongue mass, scintigraphy with 99mTc-pertechnetate for the salivary glands showed decreased uptake in the bilateral parotid and submandibular glands. 

## 3. Discussion

Lymphoma is a malignant tumor originating from the lymphoid system and is the third most common neoplasm in the head and neck. Malignant lymphomas are classified as Hodgkin’s lymphoma and NHL. NHL accounts for approximately 5% of the head and neck malignancies, and primary extranodal NHL accounts for approximately 10–20% of NHL in the head and neck [7]. In addition, autoimmune diseases of the salivary glands are usually associated with benign lymphoepithelial or lymphoproliferative lesions. Histologically, it involves the destruction of the gland cells as lymphocytes infiltrate the salivary glands and the surrounding parenchyma [8].

SS is an autoimmune disease of the salivary glands, which occurs in adults and is more common in women between 40 to 60 years of age. The incidence of NHL is 7–19 times higher in patients with SS than in normal healthy individuals. Most lymphoma occurring in patients with SS is classified as MALT lymphoma [2]. SS-associated MALT lymphoma is commonly encountered in the parotid gland. However, MALT lymphomas are rarely observed in the minor salivary glands [3,4,5,6]. To date, only five cases of histopathologically confirmed MALT lymphoma of the tongue have been reported in the English literature [6,8,9,10,11]. However, there has been no case associated with SS. In addition, only one case occurred in the dorsum of the tongue [6] and only one reported case has provided a limited representative CT image [9]. Therefore, to the best of our knowledge, this is the first report of a MALT lymphoma in the minor salivary tissue of the tongue in a patient with SS. We also provide an accurate summary of MALT lymphoma in the tongue regardless of SS through a meticulous review of the literature (Table 1).

The concept of MALT lymphoma was introduced by Isaacson and Wright in 1983. It was NHL that occurred in MALT, lymphoid tissue that is involved in antibody production and is present in the mucous membranes, which protects the epithelium of the digestive system and mucous membranes. MALT lymphoma is an extranodal marginal zone lymphoma which most commonly occurs in the gastrointestinal mucosa. However, it can occur anywhere in the mucosa throughout the body, and it can also occur in alternative extranodal sites, such as the orbit, intestine, thyroid, lung, skin, breast, salivary glands, urinary bladder, and kidney. However, it is rarely observed in the central nervous system. Pathophysiologically, MALT lymphoma involves lymphomagenesis through chronic antigenic stimulation of B-cell populations at extranodal sites. This antigen-driven concept is related to several factors, including infectivity associated with marginal zone lymphomas and autoimmune antigens seen in SS [13].

Therefore, when a patient visits the hospital for a mass in the salivary glands, including the tongue, the clinician must consider the possibility of SS-associated MALT lymphoma as the diagnosis by evaluating whether dry eyes and mouth are present along with the mass. Furthermore, it is necessary to perform additional physical, radiological, pathological, and hematological tests according to the revised classification criteria of the 2016 American College of Rheumatology/European League Against Rheumatism (ACR/EULAR) to diagnose SS [14].

In terms of significant findings other than these classification criteria, most of the radiological findings of SS-associated MALT lymphomas have been observed in the parotid gland, such as (1) the solid-cystic form is more common than the solid form on CT and MRI, and the lesion may show calcification and clear or unclear boundaries and may occur solitarily or in multiples, and (2) the parotid gland may show diffuse swelling with a honeycomb-like appearance or multiple solid cystic changes in the parenchyma [15]. In contrast, MALT lymphoma of the tongue is a very rare disease. Therefore, when radiologists encounter a well-circumscribed, enhancing tongue mass with homogeneous attenuation or signal intensity on CT and MRI, the differential diagnosis should be considered as follows: (1) a tumor of skeletal muscle origin such as rhabdomyoma or leiomyoma, (2) a tumor of minor salivary gland origin such as pleomorphic adenoma, myoepithelioma, or malignant tumors such as adenoid cystic carcinoma or mucoepidermoid carcinoma, (3) a tumor of neural origin such as schwannoma or neurofibroma, and (4) other malignancies such as tongue cancer, metastasis, and lymphoma. However, a definitive diagnosis usually requires excision or biopsy because it is difficult to differentiate these lesions on imaging studies when the size of the lesion is small. In addition, it can be helpful to evaluate coexisting abnormalities in other anatomical structures within a scanned range such as the salivary glands for a differential diagnosis, as with our case. An accurate diagnosis of MALT lymphoma can be established by histopathological examination, where the characteristic features are centrocyte-like tumor cells with irregular nuclei, follicular colonization, lymphoepithelial lesions, and plasma cells, as seen in our patient [16,17]. In immunohistochemical analysis, the tumor cells demonstrate positive results for CD19, CD20, CD22, CD79a, IgM, and BCL-2 in neoplastic cells and negative results for CD5, CD10, and cyclin D1 [18].

Patients with MALT lymphoma generally have a good prognosis, and when diagnosed, they can be cured with surgical excision only. Furthermore, chemotherapy and radiotherapy can be considered when there are limitations to the surgical treatment.

In conclusion, we described an extremely rare case of MALT lymphoma in SS patient, presenting as a painless tongue mass, with clinical, radiological, and histopathological findings. In clinical practice, the radiological and clinical findings of MALT lymphoma can be nonspecific, as shown in our case, and the rarity of SS-associated MALT lymphoma in the minor salivary gland can lead to an even more challenging diagnosis. Therefore, throughout this case report, we highlight that a meticulous radiological review with awareness of the pathophysiological association between SS and lymphoma may be greatly helpful in establishing an accurate diagnosis.

## Figures and Tables

**Table 1:** Reported cases of MALT lymphoma of the tongue based on histopathological diagnosis in the literature †.

No.	Reference	Age/Sex	Clinical Symptoms	Site Involved at Presentation	Size (cm)	IMMUNOHISTOCHEMISTRY		Sjögren’s Syndrome	Treatment	Imaging Modality
						+	−			
1	Ferry JA et al. (1996) [10]	62/F	NA	Tongue base, right false vocal cord and vallecula	NA	CD20+, CD5+, CD43+, CD23+	sIg− Cyclin-D1−	No	Conservative treatment	NA
2	Sakabe H et al. (2003) [11]	61/F	Oral discomfort, excessive thirst	Tongue base	1.2 × 1.5 × 0.5	CD20+, CD19+, HLA-DR+	CD5−, CD10−,	No	Surgical resection	NA
3	Goteri G et al. (2004) [6]	80/F	Dysphagia	Dorsum of tongue	NA	CD20+, CD5+, CD43+, CD3+	CD10−, CD23− DBA44−	No	Surgical resection	NA
4	Song JH et al. (2014) [9]	29/F	Odynophagia, underlying peripheral T cell lymphoma	Tongue base, right oropharyngeal wall	NA	CD20+ CD79a+ Bcl-2+	CD45RO− CD3−	No	Radiotherapy	Computed tomography: lobulated hypodense mass
5	Iftikhar H et al. (2016) [12]	61/M	Difficulty in swallowing, feeling of a lump in throat	Tongue base	3.2 × 2.3	CD20+, CD3+, CD5+	CD10−, CD23−, Cyclin-D1	No	Surgical excision & chemotherapy	NA

Note: this table includes only articles available in full-text form in the literature. NA, not applicable.

## Data Availability

Not applicable.
